# A synthetic three-dimensional niche system facilitates generation of functional hematopoietic cells from human-induced pluripotent stem cells

**DOI:** 10.1186/s13045-016-0326-6

**Published:** 2016-09-29

**Authors:** Yulin Xu, Wei Shan, Xia Li, Binsheng Wang, Senquan Liu, Yebo Wang, Yan Long, Ruxiu Tie, Limengmeng Wang, Shuyang Cai, Hao Zhang, Yu Lin, Mingming Zhang, Weiyan Zheng, Yi Luo, Xiaohong Yu, Jiing-Kuan Yee, Junfeng Ji, He Huang

**Affiliations:** 1 Bone Marrow Transplantation Center, The First Affiliated Hospital, School of Medicine, Zhejiang University, Hangzhou, 310012 China; 2 Department of Diabetes and Metabolic Diseases Research, City of Hope, Duarte, CA 91010 USA; 3 Center of Stem Cell and Regenerative Medicine, School of Medicine, Zhejiang University, Hangzhou, 310012 China

**Keywords:** Induced pluripotent stem cells, Hematopoietic stem cells, Niche, Three dimension, Transplantation

## Abstract

**Background:**

The efficient generation of hematopoietic stem cells (HSCs) from human-induced pluripotent stem cells (iPSCs) holds great promise in personalized transplantation therapies. However, the derivation of functional and transplantable HSCs from iPSCs has had very limited success thus far.

**Methods:**

We developed a synthetic 3D hematopoietic niche system comprising nanofibers seeded with bone marrow (BM)-derived stromal cells and growth factors to induce functional hematopoietic cells from human iPSCs in vitro.

**Results:**

Approximately 70 % of human CD34^+^ hematopoietic cells accompanied with CD43^+^ progenitor cells could be derived from this 3D induction system. Colony-forming-unit (CFU) assay showed that iPSC-derived CD34^+^ cells formed all types of hematopoietic colonies including CFU-GEMM. *TAL-1* and *MIXL1*, critical transcription factors associated with hematopoietic development, were expressed during the differentiation process. Furthermore, iPSC-derived hematopoietic cells gave rise to both lymphoid and myeloid lineages in the recipient NOD/SCID mice after transplantation.

**Conclusions:**

Our study underscores the importance of a synthetic 3D niche system for the derivation of transplantable hematopoietic cells from human iPSCs in vitro thereby establishing a foundation towards utilization of human iPSC-derived HSCs for transplantation therapies in the clinic.

**Electronic supplementary material:**

The online version of this article (doi:10.1186/s13045-016-0326-6) contains supplementary material, which is available to authorized users.

## Background

Somatic cells can be reprogrammed into a pluripotent state similar to embryonic stem cells (ESCs) by forced expression of Oct4, Sox2, Klf4, and c-Myc [1–4]. Owing to their potential to differentiate into cells of all three germ layers and their ability to expand indefinitely in culture without losing pluripotency, human-induced pluripotent stem cells (iPSCs) have become a highly attractive source of producing large-scale patient-specific cells with therapeutic potential on a variety of degenerative diseases [5–8]. Moreover, they also have important applications in drug screening and toxicology [9–11].

Transplantation of hematopoietic stem cells (HSCs) has been widely used to treat various diseases including hematopoietic and non-hematopoietic malignancies in the clinic [12, 13]. However, the conventional sources of HSCs such as cord blood, bone marrow (BM), and mobilized peripheral blood are often in short supply. HSCs derived from patient-specific iPSCs can potentially provide an unlimited supply of human leukocyte antigen-matched transplantable cells for personalized therapies [14].

Differentiation protocols are well established for the production of hematopoietic cells from pluripotent stem cells (PSCs) including both iPSCs and ESCs. Usually, embryoid body (EB) formation or co-culture with a feeder layer, such as OP9 [15–18], OP9DL1 [19, 20], S17 [21], or liver cells [22] can coax PSCs to differentiate into hematopoietic lineages. The differentiation efficiencies can be improved by adding cocktails of factors including bone morphogenetic protein 4 (BMP4) [23, 24], stem cell factor (SCF), and fms-related tyrosine kinase 3 ligand (FLT3L) [25–27]. However, the overall output of hematopoietic development is still low (0.1–2 % of CD45^+^ cells during days 8–20 of differentiation) [28]. Recent advances have improved our understanding of the cellular and molecular mechanism that regulates PSC commitment to HSCs [29–35]. However, PSC-derived hematopoietic progenitors have limited engraftment capacity [36, 37]. Thus, the efficient generation of transplantable HSCs from PSCs in vitro still remains to be a great challenge.

Cell–cell and cell–extracellular matrix (ECM) interactions are important for hematopoiesis during embryonic development [38–40]. These interactions can signal downstream pathways to regulate lineage-specific gene expression, which is required for proper tissue development. Three-dimensional (3D) self-assembling peptide scaffolds are thought to form an in vitro environment capable of simulating the in vivo milieu. Based upon this rationale, studies have reported that the relevant 3D artificial microenvironment controls stem cell behavior in vitro [41–45].

We speculated that the 3D hydrogel integrated with hematopoietic stromal cells could mimic a hematopoietic niche comprising ECM, cells of different types, and their cellular secretions. Our results showed that up to 70 % of CD34^+^ cells were generated from 3D induction systems together with CD43 expression, defining hematopoietic progenitors in human embryonic stem cell differentiation cultures [46], and mixed colony output capacity. Importantly, iPSC-derived CD34^+^ cells were capable of hematopoietic engraftment into conditioned NOD/SCID mice. Our data showed that 3D systems are an efficient method of hematopoietic differentiation. Using hematopoietic cells as a paradigm, 3D systems may be generalized to derive other functional patient-specific cell types with potential use for regenerative medicine and drug screening.

## Methods

### iPSC lines

iPSC lines, iPSC-F [27], and iPSC-EGFP (from SiDanSai Biotechnology, Shanghai, China) were used in the present study. Some of the cellular morphologies were presented from iPSC-EGFP for its GFP expression distinguishing iPSCs from the stromal cells. iPSC lines were grown on mitomycin-C-treated mouse embryonic fibroblasts (CF1) with human ESC media consisting of 80 % DMEM/F12 (Invitrogen), 20 % knockout serum replacement (KSR) (Invitrogen), 1 mM l-glutamine (Invitrogen), 1 % non-essential amino acids (NEAA) (Invitrogen), 0.1 mM β-mercaptoethanol (Invitrogen), and 4 ng/ml basic fibroblast growth factor (bFGF) (Invitrogen). Media were changed every day. Colonies were split 1:4 every 7 days with 1 mg/ml collagenase IV (Gibco).

For 3D hematopoietic induction systems, 80 % confluent iPSCs were washed in phosphate-buffered saline (PBS) free of Ca^2+^ and Mg^2+^, and incubated with 1 mg/ml collagenase IV for 10–15 min. The colonies were pipetted into small cell aggregates and transferred to a 15-ml conical tube. Cells were washed twice in PBS and prepared for 3D induction systems.

### Preparation of bone marrow, OP9, and OP9DL1

Four- to 6-week-old ICR mice were used in the study. Animal care and experiments were conducted under the approval of the Animal Care Committees of Zhejiang University. Bone marrow was isolated from the femurs. Red blood cells were lysed with 0.8 % ammonium chloride solution, and then the cells were washed, collected for 3D differentiation systems. OP9 (ATCC) and OP9DL1 (a gift from Professor Cheng Tao, State Key Lab of Experimental Hematology Institute of Hematology and Hospital of Blood Diseases, Tianjin, China) were expanded with α-minimum essential media (α-MEM) (Gibco) supplemented with 20 % fetal bovine serum (FBS; Hyclone) at 37 °C in 5 % CO_2_ atmosphere. The stromal cells were inactivated by mitomycin C treatment for 2 h (10 mg/ml) and prepared for 3D induction systems.

### 3D induction systems for iPSC differentiation into hematopoietic cells

The 3D cell culture hydrogel used in the study was custom-synthesized (Beaver Biosciences Inc., Suzhou, China), and the relevant parameters for 3D induction systems were optimized. This included cell density, the ratio of iPSCs to the stromal cells, and the sets of factors. Hydrogel was processed according to the manufacturer’s instructions. Briefly, the peptides were dissolved in distilled water at a concentration of 1 % (*v*/*w*) (pH 2–3) and treated with a ultrasonic water bath to reduce the solution viscosity and mixed with 20 % (*v*/*w*) sterile solution of sucrose for cell culture (Sigma) at a ratio 1:1 to get 0.5 % solution. iPSCs mixed with the stromal cells were washed and resuspended in 10 % (*v*/*w*) sterile solution of sucrose, and then mixed with the 0.5 % solution at a ratio 1:1 quickly at a density 1–2 × 10^6^ cells per ml. The culture media were carefully added to the well and changed twice to equilibrate the gel to physiological pH 30 min after cell seeding. The medium was then changed every 2 days until the cultures were used for analysis. Sets of factors such as 5 ng/ml BMP4, 100 ng/ml SCF, 100 ng/ml Flt3L, 20 ng/ml TPO, 2 ng/ml VEGF, 1 μM PGE2, 20 ng/ml interleukin-3 (IL-3), 20 ng/ml IL-6, 10 ng/ml EPO, and similar compounds were administrated in 3D induction systems sequentially.

### Flow cytometry analysis

Samples collected from 3D induction cultures or from the transplantation mice were isolated at time points indicated and washed in PBS supplemented with 2 % FBS. Cell clumps were removed by filtering the samples through 40-mm cell strainers, and the single-cell suspensions were incubated with mouse anti-human antigens. The following antibodies were used in the study: PE-Tra-1-85 (BD), PE-Cy5-CD34 (BD), PE-Cy7-CD34 (BioLegend), ECD-CD45 (BECKMAN COULTER), PE-CD45 (BioLegend), APC-CD45 (BioLegend), PE-Cy7-CD3 (BD), PE-Cy5-CD3 (BD), PE-Cy7-CD19 (BD), PE-Cy5-CD19 (BD), PE-Cy5-CD15 (BD), PE-Cy7-CD15 (BD), PE-CD235a (BD), PE-Cy7-CD71 (BD), FITC-CD144, FITC-CD117, FITC-CD43, PE-CD31, PE-CD38, and FITC-CD309. The incubation with the antibodies was carried out at room temperature for 30 min. The appropriate isotype IgGs (BD or BioLegend) served as controls. Cells were analyzed using a FlowCytometer FC500MCL, CXP Software, and FlowJo 7.6 Software. To ensure specificity, mouse samples, including the bone marrow, spleen, thymus, and peripheral blood samples, were stained with the same set of human antibodies as described above and the final data were reported with the background subtraction (data not shown here).

### CD34^+^ cell enrichment

An EasySep CD34 positive selection Kit (StemCell Technologies) was used to enrich CD34^+^ cells on an EasySep® Magnet device (StemCell Technologies) according to the manufacturer’s instructions.

### Colony-forming unit assay

To analyze the multi-lineage potential, 200 CD34^+^ cells were sorted from 3D induction systems and cultured in triplicate on a minimally adherent dish in Methocult 4434 (StemCell Technologies) for 14 to 16 days. The different colonies were identified and enumerated according to the colony shape, cell size, and the extent of visible cell content using an Olympus microscope. Lineage assignment was determined with Wright-Giemsa staining within the colony-forming unit (CFU) colonies by morphological analysis.

### Reverse transcriptase PCR analysis

The primer sets for reverse transcription PCR analysis are shown in Table 1. Total RNA was extracted using Trizol (Invitrogen) according to the manufacturer’s protocol and 1 μg RNA was applied to synthesize complementary DNA (cDNA) with a ReverTra Ace-α kit (Toyobo Bio-Technology, Co., Ltd, Shanghai, China). *GAPDH* was used as an endogenous control. PCR was performed in a 20 μl mixture containing 1× PCR buffer, 0.5 U of Taq DNA polymerase, 0.2 mM of each dNTP, 1.5 mM MgCl2, 0.2 μM of each primer, and 2 μl of each RT product as a template. The following program was carried out: initial denaturation for 4 min at 94 °C, 35 cycles of 94 °C for 15 s, 55 °C for *Oct4 and TAL1*, 59 °C for *MIXL1* and *BRACHYURY (T* Protein*)*, 53 °C for *GAPDH* for 35 s, 72 °C for 1 min, and then followed by 72 °C for 10 min.

**Table 1:** Primer sets for the study

Genes	Sequence (5′ to 3′) F	Sequence (5′ to 3′) R
*OCT4*	TTCAGCCAAACGACCATC	GGAAAGGGACCGAGGAGTA
*BRACHYURY*	CCTATTCTGACAACTCACCTG	CTGCGGCGTCGTACTGGCTGT
*MIXL*	AGCTGCTGGAGCTCGTCTT	AGAGAGGGGAACAGGTTTCAA
*TAL1*	GTTGCGTACGATTGTGCTCC	GCTTTCCCCCTTTTTCGCTG
*GAPDH*	AAGGTCGGAGTCAACGG	GGAAGATGGTGATGGGATT
*NANOG*	TTTGTGGGCCTGAAGAAAACT	AGGGCTGTCCTGAATAAGCAG
*GATA2*	CAGCAAGGCTCGTTCCTGTT	GGCTTGATGAGTGGTCGGT
*LMO2*	AAGCGGATTCGTGCCTATGAG	AGTTGATGAGGAGGTATCTGTCA
*RUNX1*	CTGCCCATCGCTTTCAAGGT	GCCGAGTAGTTTTCATCATTGCC
Human mitochondria	CGGAGGACAACCAGTAAGCTACCCTTTTACCATCATT	GAGCTGCATTGCTGCGTGCTTGATGCTTGTTCCTTT
Mouse mitochondria	GGCCAACTAGCCTCCATCTCATACTTCTCAATCATC	GGGATTTTACACCGGTCTATGGAGGTTTGCATGTGTAA

### Transcription factor analysis using single-cell qRT-PCR

The kinetic expression of *NANOG*, *GATA2*, *LMO2*, and *RUNX1* was detected using single-cell qRT-PCR analysis. Primer sets for the targeted genes (Table 1) were pooled to a final concentration of 0.1 μM for each primer. Individual cells were picked up directly into PCR tubes loaded with 5 μl RT-PCR master mix (2.5 μl One Step SYBR® Green Mix, 0.5 μl primer pool, 0.1 μl One Step SYBR® Green Enzyme Mix, 1.9 μl nuclease-free water) (HiScript® II One Step qRT-PCR SYBR® Green Kit) (Vazyme, Nanjing, China) in each tube. Picked cells were immediately frozen in −80 °C for at least 5 min. After brief centrifugation, the samples were immediately performed for cDNA synthesis with the following program: sequence-specific reverse transcription for 60 min at 50 °C; reverse transcriptase inactivation and Taq polymerase activation for 3 min at 95 °C; cDNA synthesis for 20 cycles of denaturing at 95 °C for 15 s, annealing and elongation at 60 °C for 15 min. cDNA products were diluted fivefold prior to analysis. For each group, 24–32 samples were sorted. According to the Ct values of *GAPDH*, eight samples for each group were selected for the analysis of the kinetic expression of the target genes with AceQ qPCR SYBR Green Master Mix kit (Vazyme, Nanjing, China). Transcription abundance was presented with △(35–Ct) values.

### Mouse transplantation assay

A NOD/SCID mice model of xenotransplantation was established as described previously [47]. Briefly, 6-week-old NOD/SCID mice were injected intraperitoneally with cyclophosphamide (CTX) 75 mg/day/kg body weight for the first 2 days. They then received 100 μg anti-mouse CD122 monoclonal antibody (eBioscience Inc.) by intraperitoneal injection on the third day. iPSC-derived hematopoietic cells were passed through a filter to remove debris, and about 500,000 cells were transferred into the conditioned NOD/SCID mouse via tail vein injection on the following day. Eight to 12 weeks after transplantation, the peripheral blood, bone marrow, spleens, livers, kidneys, intestines, and thymuses were obtained and prepared for flow cytometry and immunohistochemical assays. Erythrocytes in part of peripheral blood were lysed using ammonium chloride solution (0.8 % NH_4_Cl with 0.1 mM EDTA) (Stem Cell Technologies) when human lymphoid and myeloid cells were analyzed; the remaining cells were collected and stained with mouse anti-human antibodies as described above for flow cytometric analysis.

### Immunohistochemistry assay and H&E staining

The bones, livers, spleens, intestines, and kidneys from the transplanted mice were obtained at specified points in time and fixed in 10 % paraformaldehyde for 24 h. They were then processed for paraffin embedding. Five-to-seven-micrometer-thick sections were obtained, deparaffinized in xylene (Sinopharm Chemical Reagent Co. Ltd., China), and rehydrated in grades of ethanol. One set of tissue sections was treated with H&E staining and the other set was applied to immunohistochemical staining using anti-human CD45 antibody as described previously. Briefly, endogenous peroxidase was inactivated using 3 % hydrogen peroxide for 30 min. The slides were incubated in goat serum for 30 min to block non-specific binding of the primary antibodies. The sections were stained with rat anti-human CD45 (Abcam) for 30 min at room temperature, followed by biotinylated goat anti-rat IgG for 20 min and streptavidin peroxidase for 20 min (both from Maixin Bio, China). The antibodies were tested for specificity on murine bone marrow to exclude interspecies cross-reactivity. Positive signals were visualized with 3,3′-diaminobenzidine (DAB) chromogens.

### Human-specific mitochondria PCR amplification

To determine human cells in transplanted animals, we performed human-specific mitochondria detection with RT-PCR. Human CD45^+^ population was isolated using a BDFACSAriaII (Becton Dickinson) from the transplanted mice. Human CD45^+^ cells isolated from mice transplanted with human mobilized peripheral blood HSCs served as positive controls. Human- or mouse-specific mitochondria primers were devised as Amabile et al., described previously [48] (Table 1) which produced 1589 bp and 1250 bp length amplicon, respectively.

QIAamp® DNA Micro kit was used to purify genomic DNA from the sorted cells and the performance was carried out according to the instruments. The following PCR program was carried out: initial denaturing at 94 °C for 1 min, 45 cycles of 94 °C for 20 s, 68 °C for 2 min, 72 °C for 1 min, and then followed by 72 °C for 5 min. PCR products were analyzed by 2 % agarose gel electrophoresis, and images were captured with Gel Doc™ XR+ imaging system (BIO-RAD).

### Statistical analysis

Statistical analyses were performed using GraphPad Prism 5 software. For all experiments, values were shown as mean of the individual sample ± SEM. *P* < .05 was accepted as statistically significant.

## Results

### Efficient generation of CD34^+^ cells utilizing 3D hematopoietic niche induction systems in vitro

**Fig. 1:**
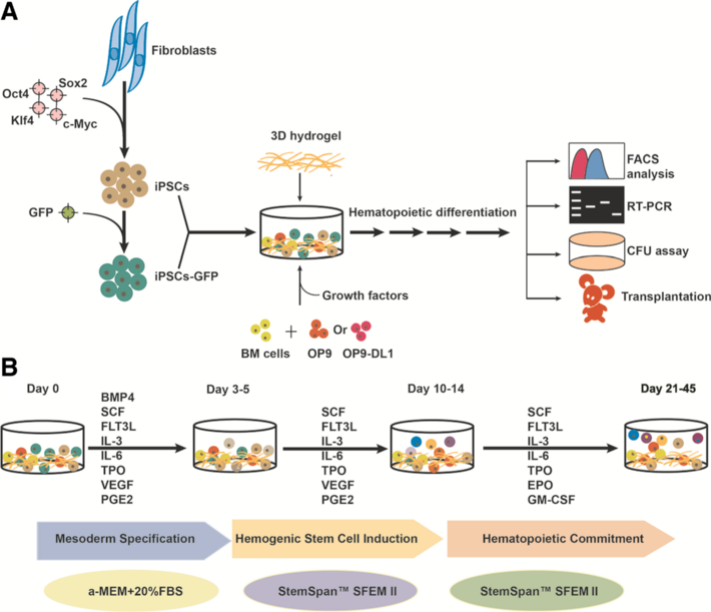
Schematic diagram of the study. **a** Schematic diagram of deriving CD34^+^ cells from human iPSCs by utilizing a synthetic 3D niche system in our study. **b** The protocol for stepwise specification of human iPSCs into hematopoietic cells

In order to develop a niche to induce hematopoietic differentiation of human iPSCs in vitro, a 3D cell culture hydrogel composed of self-assembling peptides RADA16-I (Ac-RADARADARADARADA-CONH2) was used in the study. The material formed a 3D structure and provided a synthetic ECM microenvironment for cell growth and differentiation. RADA16-I hydrogel formation by parallel β-sheet alignment of peptides was illustrated and described by Mazda Rad-Malekshahi and colleagues [49]. To further mimic the hematopoietic niche which comprises not only ECM but also supportive stromal cells and their secreted factors, hydrogel was mixed with the stromal cells of either mouse BM, or/and OP9 or/and OP9DL1 (Fig. 1a). The systems were then sequentially supplemented with sets of factors including BMP4, SCF, FLT3L, TPO, VEGF, PGE2, IL-3, IL-6, GM-CSF, G-CSF, and EPO (Fig. 1b). 3D structure would be destroyed with pipetting, and cells were free from 3D structure followed by later molecular and cellular analysis.

iPSC lines, iPSC-F [27] and iPSC-EGFP (from SiDanSai Biotechnology, Shanghai, China), were used in the present study. We first examined the morphological changes during the differentiation process in the 3D systems. Round grape-like cells resembling hematopoietic cells emerged in the differentiation cultures, and these cells formed clusters at days 10–14 in all the conditions (Additional file 1).

RT-PCR analysis showed that the expression of *BRACHYURY* (*T*), *TAL1*, and *MIXL1* were upregulated, whereas the level of *OCT4* expression declined following differentiation in the 3D systems (Additional file 2 A-D). Additionally, the kinetic expression of *NANOG*, *GATA2*, *LMO2*, and *RUNX1* was analyzed with single-cell gene qRT-PCR assay. Those gene transcript abundance was presented using △(35–Ct) values (Additional file 2 E-H). The results demonstrated that the expression of *NANOG* declined during the induction days, and the expression of *GATA2*, *LMO2*, and *RUNX1* were elevated first, and then reduced, later upregulated again, indicating a kinetic process of hematopoiesis in 3D induction systems.

**Fig. 2:**
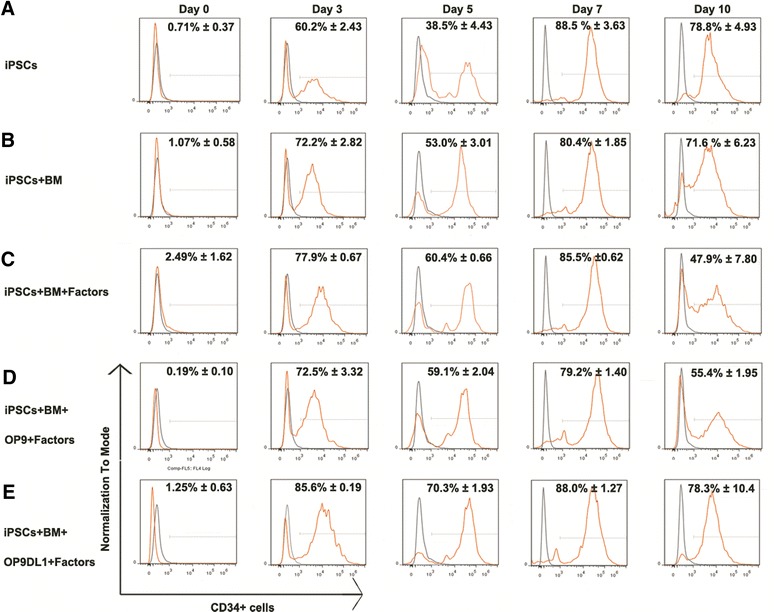
Kinetics of human CD34+ cell expression in the 3D induction systems during the differentiation for 0–10 days. The results showed that CD34^+^ cells were routinely detected in 3D induction systems, and its expression was kinetic states. From the initial induction, the percentage was low, and then began to increase. At about day 7, the peak was about 90 %, and then began to decrease. In all 3D induction cultures, peptide scaffolds fused with OP9DL1, BM plus sets of factors held better capacity to produce CD34^+^ cells during the induction phase. Data were shown as mean ± SEM, *n* = 3–6

To further examine the differentiation kinetics and efficiencies, flow cytometry analysis was performed and our results demonstrated that CD34^+^ cell population was robustly induced by all the tested 3D systems (Fig. 2).

**Fig. 3:**
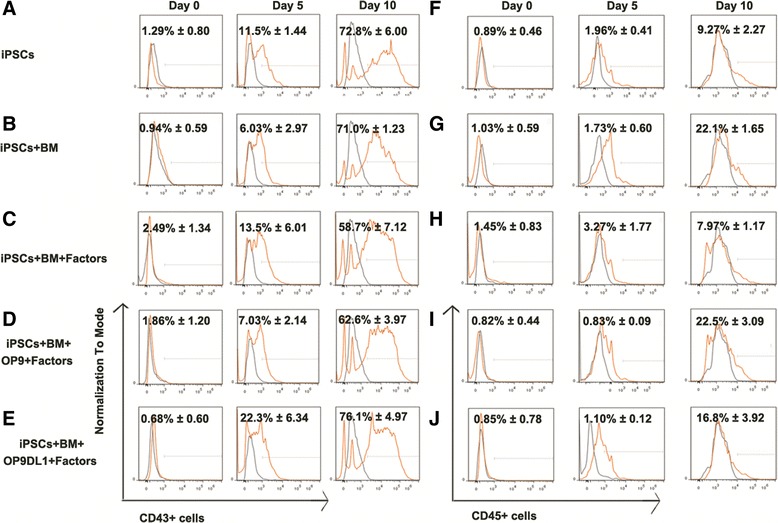
Expression pattern of the hematopoietic progenitor markers CD43 and CD45 during iPSC commitment to CD34^+^ hematopoietic cells in 3D induction systems. Kinetic expression of CD43^+^ cells (**a**–**e**) and CD45^+^ cells (**f**–**j**) during the hematopoietic cell differentiation of iPSCs in different 3D induction systems fused the stromal cells and factors with flow cytometry analysis. On the initial induction day, cultured iPSCs expressed few CD43 (day 0) or CD45 (day 0). After culture in 3D induction systems for 5 days, cells expressed about 30 % percentage of CD43^+^ but less than 5 % of CD45^+^ cells. When the induction lasted to day 10, about 70 % of the cells expressed CD43; however, the level of CD45 expression remains low about 10 %

The expression of CD43, a hematopoietic progenitor marker, and CD45, the pan-leukocyte marker, were also increased along with the induction from day 0 to day 10, respectively (Fig. 3).

**Fig. 4:**
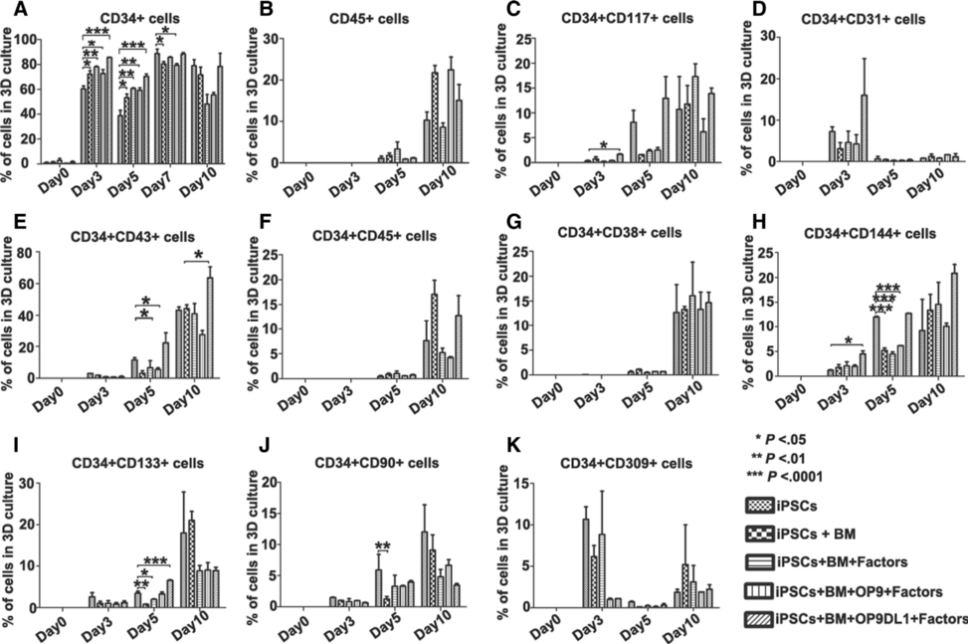
Statistical analysis of surface marker expression on developing cells during iPSC differentiation to hematopoietic cells in 3D induction systems. Cells were harvested from 3D induction cultures on the indicated days and processed for flow cytometry assay to determine the committed cell properties. Statistic analyses showed that the expression or coexpression patterns of CD34, CD45, CD117, CD31, CD43, CD38, CD144/VE-cadherin, CD133, CD90, and CD309/KDR presented on a subpopulation of different phase population, indicating the commitment of the hemangioblasts, hemogenic endothelium, hematopoietic stem cells, and hematopoietic progenitors in the cultures. Data were represented with mean ± SEM; *n* = 3

Because HSCs are generated from a specialized population of endothelial cells, known as hemogenic endothelium during embryonic development [50], we examined a few important hemogenic endothelium markers including CD144, CD133, CD90, CD31, CD309/KDR/VEGFR-2, and CD117 (SCF receptor) in our 3D induction systems (Fig. 4) [51–54]. Our results showed that these markers were co-expressed with CD34 during the differentiation process, indicating that the presence of the endothelial vascular cells, or hematopoietic stem cells, or multi-potential myeloid precursors in 3D induction cultures (Fig. 4f–j). Collectively, it suggests that the 3D systems were capable of robustly inducing stepwise differentiation of human iPSCs into hemogenic endothelium and hematopoietic progenitors. The system combining OP9DL1 in the presence of BM and the factors showed the best ability to produce CD34^+^ hematopoietic cells during the induction time from day 3 to day 10.

### Derivation of functional hematopoietic progenitors by the synthetic 3D systems

**Fig. 5:**
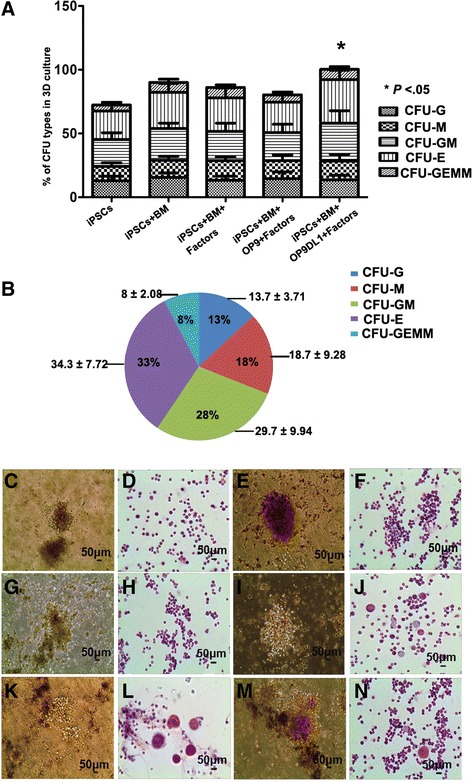
Hematopoietic colony-forming unit potential assay of CD34^+^ cells obtained from 3D induction systems. **a** Distribution of total hematopoietic colonies and colony subtypes obtained under differentiation of iPSCs in various 3D induction systems (*error bars* represented mean ± SEM; *n* = 3). Compared to no feeder control group, CFU potential of iPSC differentiating on 3D induction systems seeded with bone marrow and OP9DL1 held greater hematopoietic capacity. **b** Calculation of hematopoietic colony subtypes obtained under differentiation of iPSCs on the stromal cells derived from bone marrow and OP9DL1, indicating hematopoietic lineage forming from CD34^+^ hematopoietic cells (mean ± SEM; *n* = 3). **c**–**n** Various types of CFUs were observed as CFU-E (**c**), BFU-E (**e**), CFU-G (**g**), CFU-GM (**i**), CFU-ME (**k**), and CFU-GEMM (**m**) when CD34^+^ cells were cultured into CFU system, indicating the multi-potential differentiation capabilities of CD34+ cells derived from 3D induction systems. Morphologies of the hematopoietic lineages derived from CFUs were identified with Wright-Giemsa staining as shown in **d**, **f**, **h**, **j**, **l**, and **n**, respectively

To functionally characterize the iPSC-derived hematopoietic cells by the 3D induction systems, CFU assay was carried out for various 3D induction systems in vitro. The results were represented with hematopoietic cells derived from iPSCs carrying no EGFP (Fig. 5). The distribution of total colonies and different colony subtypes was analyzed as shown in Fig. 5a. Compared to the feeder-free groups, co-culture with OP9DL1 stromal cells plus BM resulted in a significant increase in CFU hematopoietic activity, indicating much greater hematopoietic potential for 3D system comprising OP9DL cells and BM. Representative colony subtypes, including colonies of erythroid cells (CFU-E), CFU of granulocytes (CFU-G), CFU of granulocytes and macrophages (CFU-GM), and as well as mixed colonies comprising different types of these cells (colony-forming unit comprising erythroid cells, granulocytes, megakaryocytes, and macrophages (CFU-GEMM)), were calculated for 3D system comprising OP9DL cells and BM (*n* = 3) (Fig. 5b). Various colonies from 3D induction system-derived cells showed morphology representative of CFC subtypes, including CFU-E (Fig. 5c), burst-forming units of erythroid lineages (BFU-E) (Fig. 5e), CFU-G (Fig. 5g), CFU-GM (Fig. 5i), CFU of macrophages and erythroid cells (CFU-ME) (Fig. 5k), and CFU-GEMM (Fig. 5m). Identities of erythroid cells, granulocytes, megakaryocytes, and macrophages were confirmed by Wright-Giemsa staining (Fig. 5d, f, h, j, l, n). It suggests that the 3D systems efficiently induced generation of functional hematopoietic progenitors from human iPSCs. The distribution of CFU subtypes and CFU morphology analysis from 3D induction systems indicated that the 3D induction milieu, especially using peptide scaffold fused with OP9DL stromal cells and BM plus sets of factors, efficiently supports the generation of hematopoietic progenitors from human iPSCs.

### Production of hematopoietic cell lineages from iPSC-derived CD34^+^ cells

**Fig. 6:**
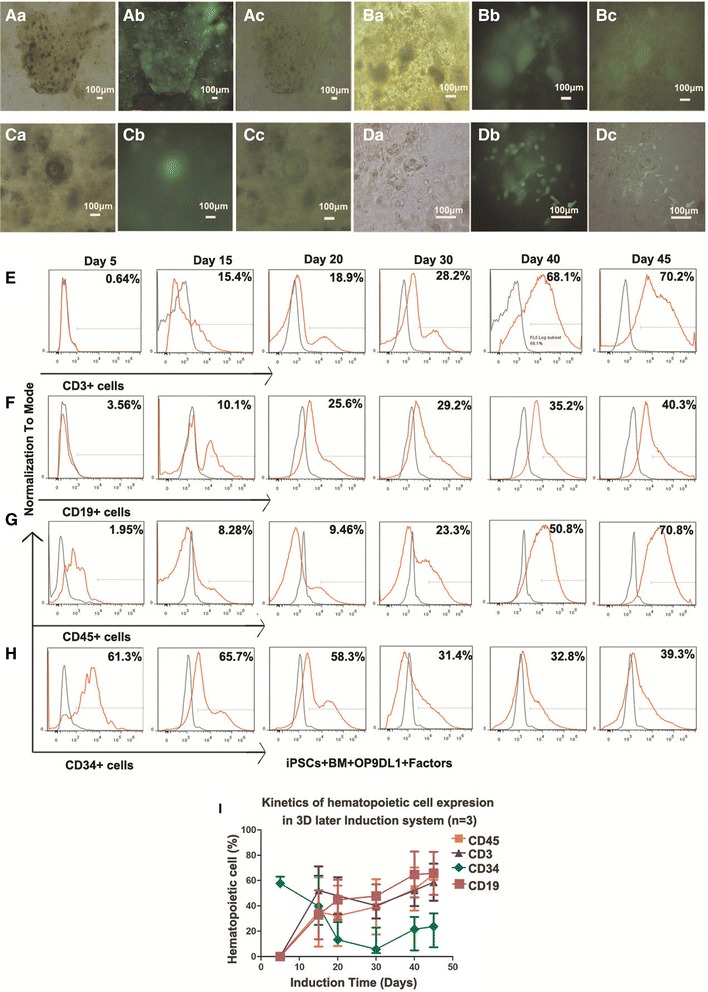
Production of mature hematopoietic cells in the following 3D culture systems. (**Aa**–**Dc**) The morphology was captured during the culture for 21–45 days. Some colonies were formed similar to CFUs in Methocult 4434 (**A**, **B**, and **C**). The relevant bright, immunofluorescent and overlapped field visions were shown in **a**, **b**, and **c**, respectively. Some cells were similar to the subtypes of lymphoid cells in morphology as shown in **Da**–**Dc**. **E**–**H** Kinetic expression of CD3^+^, CD19^+^, CD45^+^, and CD34^+^ cells to analyze the multi-lineage potential of CD34^+^ hematopoietic stem cells derived from iPSCs in vitro with flow cytometry assay. **I** Statistics analysis of CD3^+^, CD19^+^, CD45^+^, and CD34^+^ cells in 3D later induction system. *Error bars* represented mean ± SEM, *n* = 3

For the best efficiency to produce the hematopoietic cells based on the cellular markers and CFU potential analysis, the system, fused with OP9DL1 plus BM and the factors, was chosen to be presented for the multi-lineage potential analysis in vitro and in vivo. Human iPSCs were labeled with EGFP to distinguish human iPSCs from stromal cells, and green hematopoietic-like cells were observed to be derived from human iPSCs. A small proportion of CD45^+^ cells were identified in 3D induction systems during the 10–14 days of induction. This period corresponded with the peak expression of CD34^+^ hematopoietic cells. The culture media were replaced with SFEM supplemented with SCF, FLT3L, IL-3, IL-6, TPO, GM-CSF, etc., for the following 3D system culture to analyze the differentiation capacity of CD34^+^ hematopoietic cells. During the culture, human CD3^+^, CD19^+^, CD45^+^, and CD34^+^ population was monitored with flow cytometry analysis and the morphology was captured with an immunofluorescent microscope (Fig. 6). When the culture was allowed to continue for more than 3–4 weeks with a cocktail of factors including SCF, GM-CSF, TPO, and EPO, some of the cells were changed from grape-like phenotype to small round ones with different cellular sizes in morphology. At the later time points, some colonies were formed similar to CFUs in Methocult 4434 (Fig. 6A, B, C with bright, immunofluorescent, and overlapped field visions shown in a, b, and c, respectively). This may have been due to culture media supporting CD34^+^ cell commitment to hematopoietic daughter cells, and may also function in some way similar to Methocult 4434 which favors the formation of CFU. Some of the cells stretched out pseudopod-like structures which were similar to subtypes of lymphoid cells (Fig. 6D with bright, immunofluorescent, and overlapped field visions shown in Da, Db, and Dc, respectively). A very small proportion of CD3^+^, CD19^+^, and CD45^+^ cells were detected in 3D induction systems after induction for 5 days, as shown in Fig. 6E–G. With the hydrogel removal away from the system and the culture continuing, CD3^+^, CD19^+^, and CD45^+^ cells increased gradually (Fig. 6E–G). After 20 days, a population of CD3^+^, CD19^+^, and CD45^+^ cells was detected using flow cytometry analysis. The proportion increased gradually with the expression of CD34^+^ cell decrease (Fig. 6H). Kinetics of hematopoietic cells in the later culture phase were presented in Fig. 6I from day 5 to day 45 after iPSC initial induction. The data demonstrated that hematopoietic cells were generated in the culture system after hydrogel removal and iPSC-derived CD34^+^ cells held multi-lineage differentiation potential into daughter hematopoietic cells in vitro.

### The 3D system comprising BM and OP9DL1 supports the generation of hematopoietic progenitors with engraftment capacity in conditioned NOD/SCID mice

**Fig. 7:**
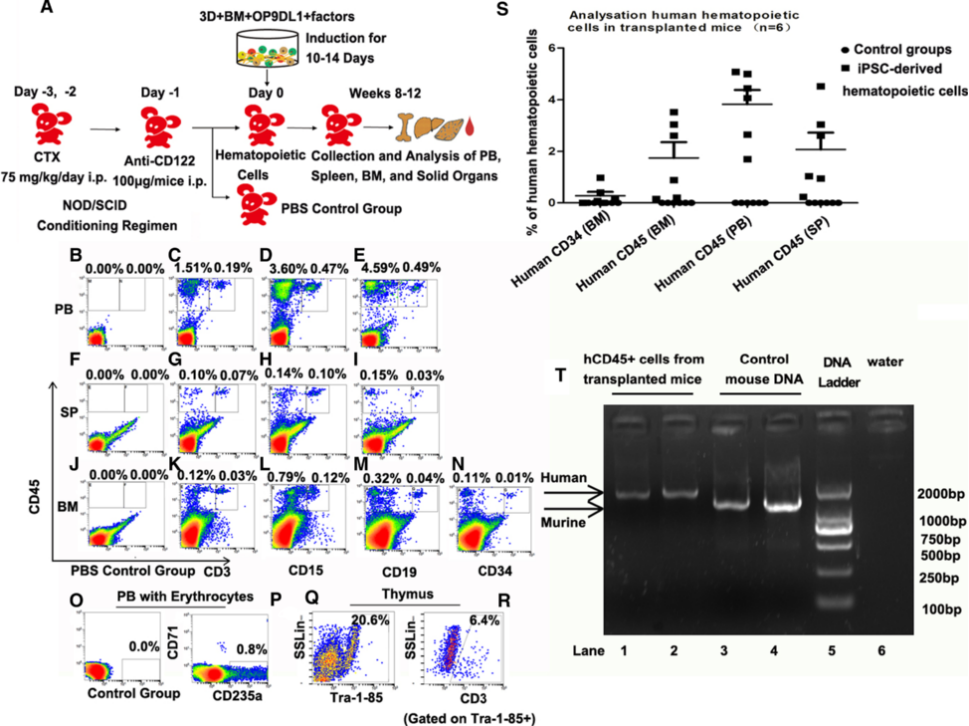
Transplantable potential analysis of iPSC-derived hematopoietic cells in conditioned NOD/SCID mice. **a** Schematic representation of xenogenic transplantation mouse model and iPSC-derived hematopoietic cell transplantation. **b**–**r** FACS analysis revealed the presence of human blood cells, myeloid cells, B cells, T cells, and CD235a + erythroid cells in the transplanted mice. The percentage of each population was indicated for a representative mouse. **b**–**e** Human CD45^+^CD3^+^ cells, CD45^+^CD15^+^ cells, and CD45^+^CD19^+^ cells were detected in the peripheral blood of the transplanted mice. **f**–**i** Human CD45^+^CD3^+^ cells, CD45^+^CD15^+^ cells, and CD45^+^CD19^+^ cells were present in the spleens of the animals. **j**–**n** Human CD45^+^CD3^+^ cells, CD45^+^CD15^+^ cells, and CD45^+^CD19^+^ and CD45^+^CD34^+^ cells were present in the bone marrow. **o**, **p** The CD235a^+^ population was shown as the percentage compared with the total blood cell number. **q**, **r** CD3^+^ cells, gated on Tra-1-85^+^ cells, were present in thymuses of the transplanted mice, indicating that human T cells had entered the thymuses. **s** Different blood populations were analyzed in the transplanted mice. The value was the mean ± SEM of six independent experiments. **t** The results of RT-PCR showed that human-specific mitochondria production was detected in the cells sorted from the transplanted animals with human CD45 antibody staining. Lanes 1–2: amplification of human CD45^+^ cells sorted from the transplanted mice using human-specific primers, indicating that human hematopoietic cells presented in the transplanted animals. Lane 3–4: demonstration of the mouse DNA control using mouse-specific primers. Lane 5: DNA ladder markers. Lane 6: negative control with water. *CTX* cyclophosphamide, *i.p.* intraperitoneal, *PB* peripheral blood, *BM* bone marrow, *SP* spleen

To further investigate the engraftment potential of iPSC-derived hematopoietic progenitors induced by the 3D induction system integrating BM and OP9DL1, the cells were transplanted into the immune-compromised NOD/SCID mice as shown by the schematic diagram similar to the transplantation protocol used in the clinic [55] (Fig. 7a). Cyclophosphamide (CTX) was used to suppress the immune response of the recipients together with anti-mice mono-antibody CD122 as previously shown [47]. Based upon the induction efficiency and the multi-potency of the cells, hematopoietic cells from the systems seeded with BM and OP9DL1 plus factors were used for the transplantation experiments. Eight to 12 weeks after transplantation, the peripheral blood, bone marrow, spleens, livers, intestines, kidneys, and thymuses were obtained and prepared for human hematopoietic cell lineage analysis by flow cytometry and immunohistochemistry. Our results showed that human hematopoietic cell lineages, including CD45^+^CD3^+^ (Fig. 7c, a shows control samples of peripheral blood with erythrocytes lysed), CD45^+^CD15^+^ (Fig. 7d), CD45^+^CD19^+^ cells (Fig. 7e), CD45^+^CD3^+^, CD45^+^CD15^+^, and CD45^+^CD19^+^ cells, were detected in the spleens of the recipient mice (Fig. 7f–i). Human CD45^+^CD3^+^, CD45^+^CD15^+^, CD45^+^CD19^+^, and CD45^+^CD34^+^ cells were also present in the BM (Fig. 7j–n). CD71^−^CD235a^+^ erythroid cells (Fig. 7p, o shows control samples of peripheral blood with erythrocytes) were detectable in the peripheral blood of the recipient mice. Tra-1-85^+^CD3^+^ cells were present in thymuses (Fig. 7q, r), indicating that human T cells had entered the thymuses. Quantitative analyses of different blood populations in the transplanted mice were performed as shown in Fig. 7s. The results of RT-PCR showed that human-specific mitochondrion DNA appeared in the cells sorted from the transplanted recipients with human CD45^+^ antibody staining, confirming the presence of human cells in the recipient mice (Fig. 7t).

**Fig. 8:**
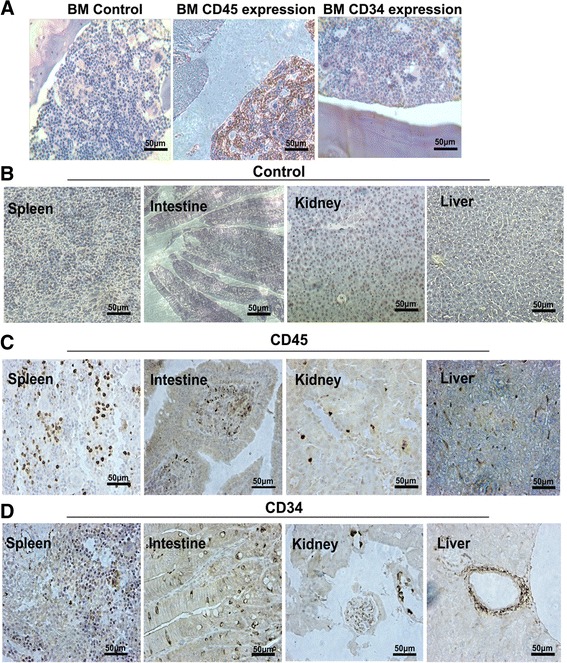
Human cell reconstitution of the organs of the transplantation recipients. **a** An immunohistochemical assay demonstrated that human CD45^+^ and CD34^+^ were present in the bone marrow of the transplanted mice. **b** Control samples was stained with human CD45 antibody for the control recipients transplanted with PBS. **c**, **d** Human CD45^+^ and CD34^+^ cells were detected in the livers, spleens, intestines, and kidneys of the transplanted mice

The presence of human hematopoietic cell lineages in the recipients indicated that iPSC-derived CD34^+^ cells from 3D induction systems in vitro can reconstitute the blood and immune systems of the conditioning recipients. It suggests that our 3D systems induced the generation of HSC-like cells. A predominance of human T lymphoid cells in NOD/SCID recipients was observed, which was most likely due to the effect of OP9DL1. CD3^+^ T cells, CD15^+^ myeloid cells, and CD19^+^ B cells were found in the peripheral blood, bone marrow, and spleens. CD71^−^CD235a^+^ cells were also detected in peripheral blood, suggesting that 3D-derived CD34^+^ cells can differentiate into mature hematopoietic cells including erythroid cells after engraftment. Immunohistochemical analysis further demonstrated that, in contrast to the control groups (Fig. 8b), human CD45^+^ cells (Fig. 8a, c) and CD34^+^ cells (Fig. 8a, d) were present in the bones, spleens, intestines, kidneys, and livers of the transplanted mice, respectively. It was interesting that human CD34+ cells was especially labeled in the periductal tissue in the liver and kidney, which might indicate that human CD34^+^ cells took a function in the mouse blood vessel formation. This is the first study to show that human iPSC-derived CD34^+^ hematopoietic cells induced from a simulated niche in vitro are capable of multi-lineage reconstitution when transplantqied into immunodeficient mice.

## Discussion

Although much knowledge about hematopoietic development from PSCs has been gained in the past decades, low efficiency and dysfunction of the derived cells remain to be the important issues. Previous studies have reported that CD34^+^ cells from human PSCs showed little or no engraftment potential in the transplanted recipients [14, 56–59]. In 2008, Ledran and colleagues reported that co-culturing human ESCs on primary aorta-gonad-mesonephros (AGM) stroma in the presence of cytokines allowed the derivation of about 16 % CD34^+^ cells capable of primary hematopoietic engraftment. However, a robust multi-lineage reconstitution was not detected in the transplanted immunocompromised mice [28]. In 2013, two research groups reported the generation of transplantable CD34^+^ cells from iPSCs using in vivo teratoma formation method [59, 60]. However, the percentages of CD34^+^ cells obtained from the teratomas were below 0.1 % in both studies. Thus, the efficient generation of transplantable CD34^+^ cells from human PSCs in vitro is still challenging.

We speculated that the difficulty of producing transplantable CD34^+^ hematopoietic cells in vitro might be due to the non-physiologic hematopoietic niche in the previous induction strategies. Hematopoietic cells derived from the suboptimal induction systems might be deficient in homing. Therefore, the cells might either lose stem cell function or be attacked by the residual immune cells in the recipients before homing to the tissue with the appropriate supporting niche.

In the current study, a simulated physical iPSC hematopoietic differentiation niche was developed: a 3D extracellular matrix seeded with mouse BM and the stromal cell lines OP9 or OP9DL1 in the presence of growth factors. The simulated niche systems were used to study the effects of iPSC differentiation into hematopoietic cells. The results showed that CD34^+^ cell populations were robustly generated in all 3D induction systems, and the system seeded with BM and OP9DL1 showed the greatest hematopoietic potential. The highest efficiency was approximately 90 % of CD34^+^ cells in the 3D system combining OP9DL1 and BM in presence of growth factors, which to our knowledge was among the most efficient reported to date. High induction efficiencies were also detected in other 3D induction systems, indicating that our 3D niche systems could efficiently drive iPSC differentiation into CD34^+^ cells. CD34^+^ cell populations from 3D induction systems were confirmed to have hematopoietic cell potential. The hematopoietic transcription factors *TAL1*, *MIXL1*, *GATA2*, *LMO2*, and *RUNX1* were confirmed to be expressed kinetically during the differentiation process. Additionally, the developmental sequence of iPSC-hematoendothelial precursors (HEPs, CD31^+^CD34^+^CD45^−^), hematopoietic progenitors (CD34^+^CD43^+^ CD45^−^), and bona fide CD45^+^ blood cells was presented in 3D induction systems. The derived cells formed all types of CFUs including CFU-G, CFU-GM, CFU-M, CFU-E, BFU-E, and CFU-GEMM. Human CD45^+^ cells, CD3^+^ cells, CD15^+^ cells, and CD19^+^ cells were detected in the following culture systems, demonstrating that CD34^+^ cells are multi-potent and can form all hematopoietic cell lineages.

In mouse transplantation assays, the entire cell population from the 3D induction system co-cultured with BM and OP9DL1 was used in the presence of growth factors. Rapid homing to the BM niche is critical to the survival of transplanted HSCs and subsequent function in recipients. Engraftment capacity of iPSC-derived CD34^+^ hematopoietic cells was confirmed by a definitive presence of human hematopoietic cell lineages in the peripheral blood, BM, spleen, and even thymuses, indicating successful engraftments into conditioned NOD/SCID mice. Multi-lineage reconstitution including human CD3^+^CD45^+^ cells, CD19^+^CD45^+^ cells, CD15^+^CD45^+^ cells, and mature CD71^−^CD235a^+^ erythrocytes were readily detectable in the peripheral blood of the transplanted mice. CD34^+^CD45^+^ cells, CD3^+^CD45^+^ cells, CD19^+^CD45^+^ cells, and CD15^+^CD45^+^ cells were detected in the BM and spleens of the transplanted mice. A human Tra-1-85^+^CD3^+^ double positive population has been produced in thymuses, indicating that T cell progenitors differentiated from true HSCs [51]. The results of immunohistochemical examination showed the presence of human CD45^+^ cells in the BM, livers, spleens, intestines, and kidneys of the transplanted mice.

In our study, we compared the differentiation protocol efficiency in 2D (co-culture of iPSCs on OP9DLL +/− BM cells) versus 3D system. However, the efficiency from 2D system was very low, and human cells were not detected in the transplanted mice (data not shown here). Therefore, we might infer that the 3D induction system loaded with BM, which contained the osteoblastic and the vascular cells, might exert the molecular and cellular effect during PSC commitment to HSC, or later on HSC maintaining. RNASeq arrays would be carried out to determine the real biology of iPSC-derived hematopoietic cells using 3D induction systems in the future.

The present study has indicated, for the first time, that efficient commitment of human iPSCs to transplantable CD34^+^ hematopoietic cells in vitro is feasible. We believe that this novel system, which involves 3D ECM combined with relevant stromal cells from the niche in the presence of inducers or cytokines, will be a robust method to produce functional human hematopoietic progenitor cells with therapeutic potential for clinical applications. It may open up a new avenue in cell transplantation, disease treatment, and regenerative medicine.

## Conclusions

In this study, we developed a synthetic 3D hematopoietic niche system using hydrogel seeded with bone marrow (BM)-derived stromal cells and growth factors to induce functional hematopoietic cells from human iPSCs in vitro. CD34+ hematopoietic cells accompanied with CD43+ and CD45+ progenitor cells could be efficiently generated in 3D induction systems. Critical transcription factors associated with hematopoietic development were expressed during the differentiation process. Mixed CFU assay indicated that iPSC-derived CD34+ cells held multi-lineage commitment potential. Especially, iPSC-derived hematopoietic cells gave rise to both lymphoid and myeloid lineages both in vitro and in vivo. Our study underscores the importance of a synthetic 3D niche system for the derivation of transplantable hematopoietic cells from human iPSCs in vitro thereby establishing a foundation towards utilization of human iPSC-derived HSCs for transplantation therapies in the clinic.

## Additional files

Additional file 1: Growth kinetics of iPSCs seeded in various 3D hematopoietic induction milieu from day 0 to day 10-14. iPSCs were digested with collagenase IV, pipetted gently into small cell aggregations, and then seeded in 3D induction culture. The morphology was captured at indicated time points post seeding. The morphology on day 0 was shown in fig A, D, G, J and M representative of different 3D hematopoietic induction milieu. After 3 days, the formation of a blast colony from iPSC mass was detected as shown in fig B, E, H, K, and N. During induction for 10–14 days, round grape-like cells appeared in 3D induction systems as shown in C, F, I, L, and O. There was no obvious disparity in morphology among various 3D induction systems. (JPG 522 kb)
Additional file 2: Characterization of the committed cells from iPSCs in 3D induction systems. (A) The relative expression of the pluripotent marker *OCT4* decreased gradually during the induction phase. (B) The expression of mesoderm transcription factor *BRACHYURY* was analyzed. (C) Enhanced expression of *TAL-1* was detected. (D) The hematopoietic cell markers *MIXL1* was significantly elevated during iPSC commitment to CD34+ cells. The values were the mean ± SEM of 3 independent experiments. (E-H) Kinetic expression of *NANOG*, *GATA2*, *LMO2,* and *RUNX1* in 3D induction systems using single-cell gene expression analysis. Transcript abundance are calculated with △(35–Ct) values. *N* = 8. Error bars represented standard error of mean for each sample. (JPG 508 kb)

## Abbreviations

HSCs: Hematopoietic stem cells
iPSCs: Induced pluripotent stem cells
BM: Bone marrow
CFU: Colony-forming unit
ESCs: Embryonic stem cells
PSCs: Pluripotent stem cells
EB: Embryoid body
BMP4: Bone morphogenetic protein 4
SCF: Stem cell factor
FLT3L: fms-related tyrosine kinase 3 ligand
ECM: Cell–extracellular matrix
3D: Three-dimensional
KSR: Knockout serum replacement
NEAA: Non-essential amino acids
bFGF: Basic fibroblast growth factor
PBS: Phosphate-buffered saline
FBS: Fetal bovine serum
CFU-E: Colony-forming unit of erythroid cells
CFU-G: Colony-forming unit of granulocytes
CFU-GM: Colony-forming unit of granulocytes and macrophages
CFU-GEMM: Colony-forming unit comprising erythroid cells, granulocytes, megakaryocytes, and macrophages
HEPs: Hematoendothelial precursors
