# Supplementary figures and images for: A synthetic three-dimensional niche system facilitates generation of functional hematopoietic cells from human-induced pluripotent stem cells
Source: J Hematol Oncol. 2016 Sep 29;9:102. doi: 10.1186/s13045-016-0326-6 (PMC5043527; doi:10.1186/s13045-016-0326-6)

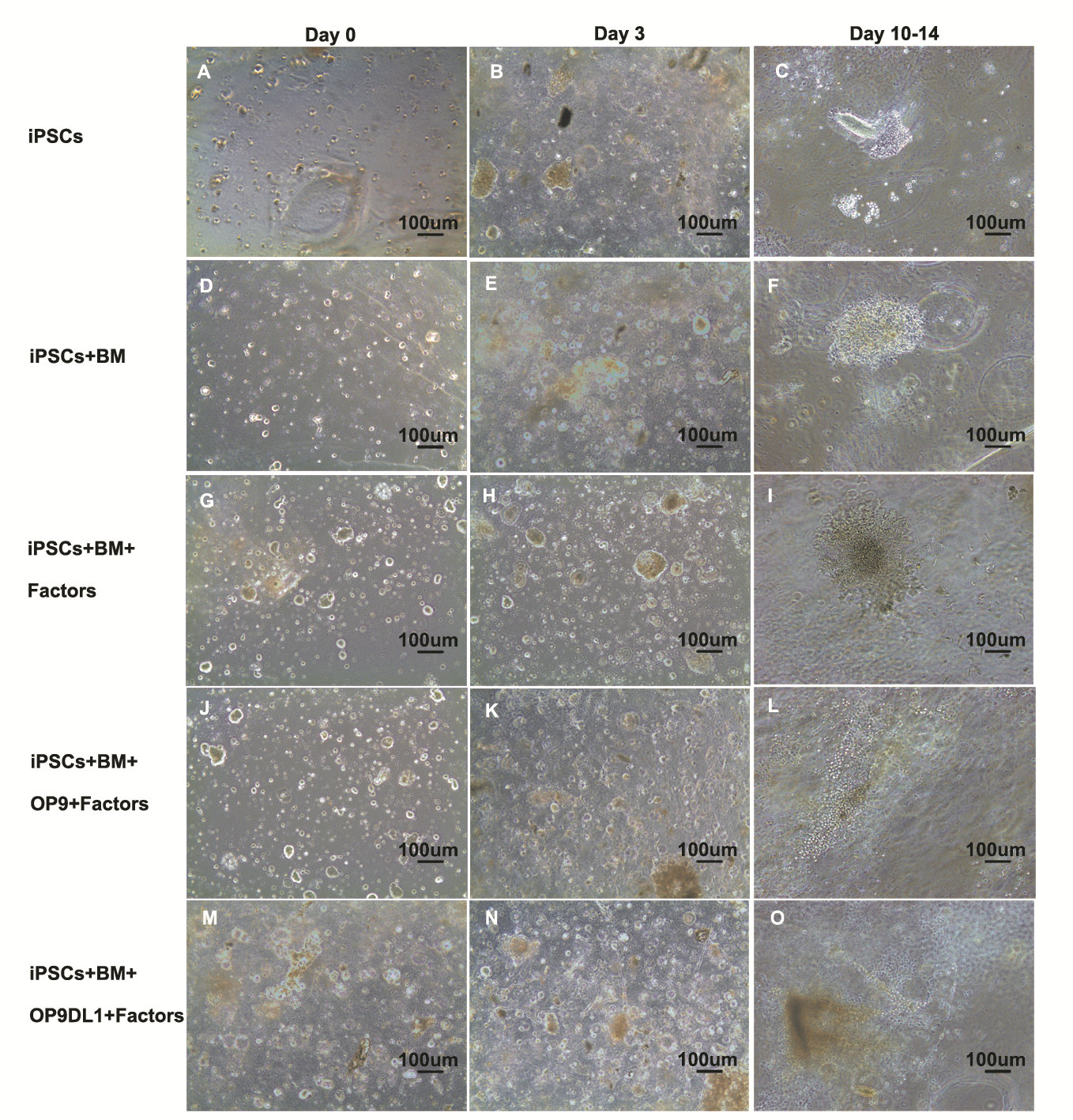

Supplement: Additional file 1: — Growth kinetics of iPSCs seeded in various 3D hematopoietic induction milieu from day 0 to day 10-14. iPSCs were digested with collagenase IV, pipetted gently into small cell aggregations, and then seeded in 3D induction culture. The morphology was captured at indicated time points post seeding. The morphology on day 0 was shown in fig A, D, G, J and M representative of different 3D hematopoietic induction milieu. After 3 days, the formation of a blast colony from iPSC mass was detected as shown in fig B, E, H, K, and N. During induction for 10–14 days, round grape-like cells appeared in 3D induction systems as shown in C, F, I, L, and O. There was no obvious disparity in morphology among various 3D induction systems. (JPG 522 kb) [file 13045_2016_326_MOESM1_ESM.jpg]

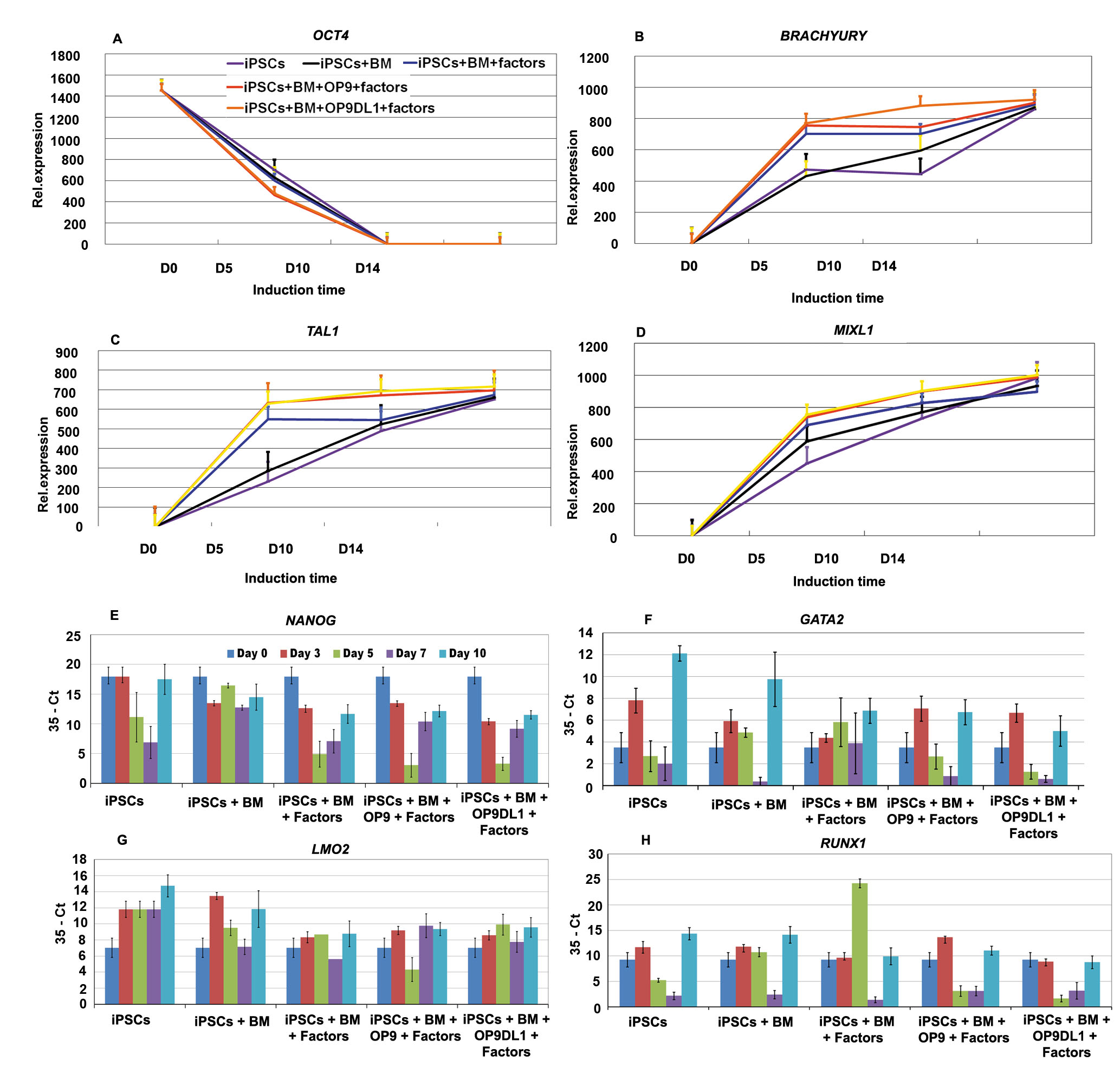

Supplement: Additional file 2: — Characterization of the committed cells from iPSCs in 3D induction systems. (A) The relative expression of the pluripotent marker OCT4 decreased gradually during the induction phase. (B) The expression of mesoderm transcription factor BRACHYURY was analyzed. (C) Enhanced expression of TAL-1 was detected. (D) The hematopoietic cell markers MIXL1 was significantly elevated during iPSC commitment to CD34+ cells. The values were the mean ± SEM of 3 independent experiments. (E-H) Kinetic expression of NANOG, GATA2, LMO2, and RUNX1 in 3D induction systems using single-cell gene expression analysis. Transcript abundance are calculated with △(35–Ct) values. N = 8. Error bars represented standard error of mean for each sample. (JPG 508 kb) [file 13045_2016_326_MOESM2_ESM.jpg]
